# Comparison of three methods for determining anti-thyrotropin receptor antibodies (TRAb) for diagnosis of Graves’ disease: a clinical validation

**DOI:** 10.1515/almed-2021-0015

**Published:** 2021-03-02

**Authors:** Ramona A. Silvestre, Alejandro Almería Lafuente, Lucía Jiménez-Mendiguchía, Ana García-Cano, Rubén Romero López, Belén García-Izquierdo, Cristina Pardo de Santayana, Pedro Iglesias, Juan J. Diez, Ignacio Arribas Gómez, Francisco A. Bernabeu-Andreu

**Affiliations:** Service of Biochemistry and Clinical Biochemistry, Puerta de Hierro Majadahonda University Hospital, Madrid, Spain; Service of Endocrinology and Nutrition, Puerta de Hierro Majadahonda University Hospital, Madrid, Spain; Service of Clinical Biochemistry, Ramón y Cajal University Hospital, Madrid, Spain

**Keywords:** anti-thyrotropin receptor antibodies (TRAb), chemiluminescence, clinical validation, Graves’ disease, immunofluorescence

## Abstract

**Objectives:**

Graves’ disease is secondary to the presence of anti-thyrotropin receptor antibodies (TRAb), which stimulate thyroid hormones. TRab determination is crucial for etiological diagnosis. The objectives of this study were (i) to compare two methods for determining TRab by chemoluminiscence vs. standard TRACE-immunofluorescence; (ii) to determine the diagnostic validity of the three methods.

**Methods:**

A retrospective study in 194 patients with a TRAb determination request. TRAb were determined by immunofluorescence (Kryptor, ThermoFisher) and chemiluminescence (Immulite, Siemens and Maglumi, Snibe). Clinical validation: medical records were reviewed and categorized according to thyroid function. Statistical analysis: Differences in *quantitative variables* were assessed by intraclass correlation coefficient, Bland–Altman plot, and mean differences (mD). Qualitative variables were dichotomized by cut-off points; Kappa coefficient was calculated. Correlations were evaluated by Pearson’s coefficient and Passing-Bablok regression analysis. The diagnostic validity of the three methods was investigated.

**Results:**

Kryptor-Immulite: mD: 1.2 (95%CI: −16 to >18). Passing-Bablok: Constant error (95%CI: −0.8349 to −0.5987). Proportional error (95%CI: 0.7862–1.0387). ICC: 0.86 (95%CI: 0.82–0.89). Kappa coefficient: 0.68 (95%CI 0.59–0.78). Kryptor-Maglumi: mD: −0.3 (95%CI: −12 to >12). Passing-Bablok: Constant error (95%CI: −0.7701 to >0.1621. Proportional error (95%CI: 0.8571 to 1.3179. ICC: 0.93 (95%CI: 0.89–0.97). Kappa coefficient: 0.53 (95%CI: 0.32–0.74). Diagnosis of Graves’ disease was confirmed in 113 patients (Kryptorf showed better specificity and positive predictive value, whereas Immulite demonstrated better sensitivity and negative predictive value).

**Conclusions:**

The three methods have a good diagnostic performance for Graves’ disease, with superimposable results on Bland–Altman plot. Interchangeability was not confirmed on the regression and agreement analysis, with the presence of biases.

## Introduction

Graves’ disease (GD), the most common cause of hyperthyroidism, is an autoimmune disease of the thyroid gland caused by the presence of anti-thyrotropin receptor antibodies (TRab). The activation of this receptor by TRab binding may stimulate the secretion of thyroid hormones and induce the development of hyperthyroidism. In this case, inhibition of thyrotropin secretion (TSH) by the negative thyroid hormone feedback mechanism is not accompanied by decreased hormone synthesis and secretion, but it is the body itself that stimulates the thyroid gland activity [1], [2]. Although the presence of clinical signs and symptoms, and TSH, free thyroxine (T4L) and free triiodothyronine (T3L) secretion are confirmatory of a diagnosis of hyperthyroidism, it is also important to determine the presence of TRAb, an immunological mechanism. In addition, TRAb plays a crucial role in the follow-up of patients with GD and the prediction of relapse. Reduced or absence of TRAb concentrations confirm disease remission and offer guidance about the optimal timing for drug therapy withdrawal. In addition, there are studies showing that TRAb determination vs. thyroid scintigraphy for the diagnosis of GD reduce health costs by 47% and accelerates diagnosis by 46% [3].

GD patients also exhibit the presence of other auto-antibodies such as anti-thyroglobulin (anti-Tg), anti-peroxidase (anti-TPO) [4], [5], and neutral antibodies [6], which may reduce specificity in the diagnosis of GD based on biochemistry.

There are a variety of methods currently available for determining TRAb [2], [7], [8]. In GD patients, there are reports of the presence of antibodies that specifically bind the TSH receptor. However, these antibodies are not capable of stimulating thyroid hormone secretion, but they block its receptor instead [9]. The presence of the two types of antibodies has been described in other patients [10], [11]. The balance between stimulating and blocking antibodies determines the status of thyroid function; therefore, the technique used to measure TRAb should be able to discriminate between thyroid-blocking and thyroid-stimulating antibodies [7], [12].

The activation of the TSH receptor can be assessed through biological tests measuring levels of cyclic adenosine monophosphate (CAMP), which is secreted as a result of the activation of the adenylate cyclase to which the TSH receptor is coupled. However, this method is scarcely used in clinical laboratories. A clinical approach emerges as an alternative to the biological model.

In our laboratory, the presence of TRAb is routinely tested by immunofluorescence based on TRACE (time-resolved amplified cryptate emission) technology (Kryptor, Thermo Fisher Scientific) [13], [14]. The objectives of this study were: (i) to compare two chemiluminescence-based systems for TRAb determination vs. the local standard method; (ii) to determine the diagnostic validity of the three systems, as compared to the gold-standard based on clinical criteria widely used for the diagnosis of GV [15], [16], [17].

## Materials and methods

A retrospective, observational cross-sectional study was performed. Data were extracted from medical records between September and December 2019.

### Patients and samples

We included 194 samples sent to our laboratory for TRAb determination between January 2016 and February 2018. A review was performed of the medical records of the patients included in the study, who were classified according to their thyroid function status, namely: (i) normal function or euthyroidism, associated with normal serum TSH concentrations (0.35–5.0 µUI/mL) and free T4 (0.7–1.98 ng/dL); (ii) hypothyroidism, defined as low serum free-T4 concentrations; and (iii) hyperthyroidism, associated with low serum TSH concentrations and elevated levels of free T4 and/or free T3 (2.3–4.2 pg/mL); (iv) subclinical hypothyroidism (high serum TSH concentrations and normal free T4); and (v) subclinical hyperthyroidism (low serum TSH concentrations and normal free T3 concentrations).

Diagnosis of GD was established based on clinical parameters (signs and symptoms of hyperthyroidism, presence of ophthalmopathy, pretibial myxedema), imaging studies (thyroid ultrasonography and scintigraphy) and biochemistry (frank hyperthyroidism or subclinical hyperthyroidism with positive thyroid autoimmunity) [16]. These criteria are the gold-standard methods for the diagnosis of GD [16], [17]. In case scintigraphy (or ultrasonography) results were not consistent with antibody results, the presence of diffusely hyperenhancing goiter on scintigraphy is confirmatory of GD diagnosis, even in patients with negative TRAb, which may exceptionally occur in GD patients [18], [19]. Other clinical variables can also be considered in the diagnosis of GD such as time from onset of the disease, sex, age, presence of concomitant auto-immune diseases, long-term therapeutic response to antithyroid agents or the presence of diffuse goiter on palpation.

Blood was centrifuged at 1,800 × *g* to separate serum, which was stored at −80 °C for later analysis. The study was approved by the Institutional Review Board (IRB) of Puerta de Hierro Majadahonda University Hospital.

TRAb concentrations were measured using three systems: the standard system used in our laboratory (immunofluorescence, Kryptor Compact Plus, ThermoFisher, hereinafter Kryptor) and another two systems based on chemiluminescence, more specifically, the Immulite 2000 analyzer (Siemens Healthineers) (hereinafter, Immulite) and the Maglumi 800 analyzer (Snibe) (hereinafter, Maglumi).

Kryptor is based on TRACE^TM^ technology, which measures the late signal emitted by an immunocomplex. This technology is based on the transfer of nonradioactive energy from a donor (europium cryptate) to an acceptor molecule (XL665).

According to the manufacturer, Kryptor has a limit of detection of 0.27 IU/L, a sensitivity of 0.82 IU/L and a limit of quantification of 0.89 IU/L. Intra-analytical coefficients of variance are <10% for concentrations >1.2 IU/L, and <12% for concentrations between 1 and 1.2 IU/L. Internalytical CV are 10% for concentrations >2 IU/L and <18% for concentrations between 1 and 2 IU/L.

The measurement method of Immulite is based on a chemiluminiscence immunoassay that employs a pair of recombinant human TSH receptor chimeras. The limits of detection and quantification of Immulite as reported by the manufacturer are 0.06 IU/L and 0.10 IU/L, respectively.

Finally, the third laboratory system is an indirect non-enzymatic chemiluminiscence immunoassay (CLIA system) (Maglumi). According to the data provided by the manufacturer, its sensitivity is 2.5 IU/L, intra-assay CV are <8% (at low concentrations) and <5% (at high concentrations), whereas inter-assay CV is <8%.

### Statistical analysis

Descriptive analysis of quantitative variables includes mean values and standard deviation for normally distributed variables, and median and interquartile ranges for variables not with normal distribution.

Cross-comparison of the three systems was performed based on intraclass correlation coefficient (ICC) and Bland–Altman plot for quantitative variables. Agreement between qualitative dichotomous variables was assessed using Kappa coefficient. For dichotomization, we used the cut-off points provided by the manufacturer: 1.8 IU/L for Kryptor, 1.5 IU/L for Maglumi and 0.55 IU/L for Immulite. These measures were used to assess the level of agreement. In addition, Pearson’s coefficient of variation and Passing-Bablok regression analysis were also used.

The diagnostic validity of the three methods was assessed on the basis of sensitivity (S), specificity (Sp), positive and negative predictive values (PPV, NPV), positive and negative likelihood ratios (LR+ and LR−) and areas under the ROC curve (AUC).Where possible, 95% confidence intervals (CI) were included in the estimated parameters. All statistical analyses were performed using the Medcalc (MedCalc^®^ Version 11.4.2.0) software package.

## Results

Table 1 shows descriptive data for the study population (n=194), which was mostly composed of women (n=156). GD was confirmed in 113 of the 194 subjects included in the study. Of the 113 patients with GD, the Kryptor analyzer identified 86 patients with positive TRAb (above the cut-off point). Of the 18 euthyroid patients with GD, 17 had positive TRAb results. In the remainder of patients, TRAb positivity was <1%.

**Table 1:** Descriptive data of the study population.

	Women		Men	
n (%)	156	(80)	38	(20)
Age, years (x SD)	49	(16)	52	(21)
Euthyroidism	69	(35)	15	(8)
Graves’ disease, n (%)	29	(15)	6	(3)
Hyperthyroidism	73	(38)	20	(10)
Graves’ disease, n (%)	59	(19)	13	(7)
Multinodular goiter, n (%)	3	(1)	1	(<1)
Toxic adenoma, n (%)	2	(1)	1	(<1)
Other, n (%)	9	(5)	5	(2)
Hypothyroidism, n (%)	14	(7)	3	(1)
Graves’ disease, n (%)	4	(2)	2	(1)
Hashimoto disease	4	(2)	1	(<1)
Other, n (%)	6	(3)	0	(0)

% calculated with respect to the total (n=194).

Table 1 shows the underlying diseases identified, with multinodular goiter defined as the presence of nodular disease on ultrasound with nodular enhancement on scintigraphy and hyperthyroidism; toxic adenoma was defined as the presence of hyperenhancing thyroid nodules on scintigraphy with or without functional suppression of the rest of the thyroid gland associated with hyperthyroidism. The “Other” category includes silent thyroiditis, subacute thyroiditis, postpartum thyroiditis, thyrotoxicosis factitia, autoimmune chronic thyroiditis, alemtuzumab-induced hyperthyroidism, amiodarone-induced hyperthyroidism, and toxic multinodular goiter induced by iodine contrasts.

**Figure 1:**
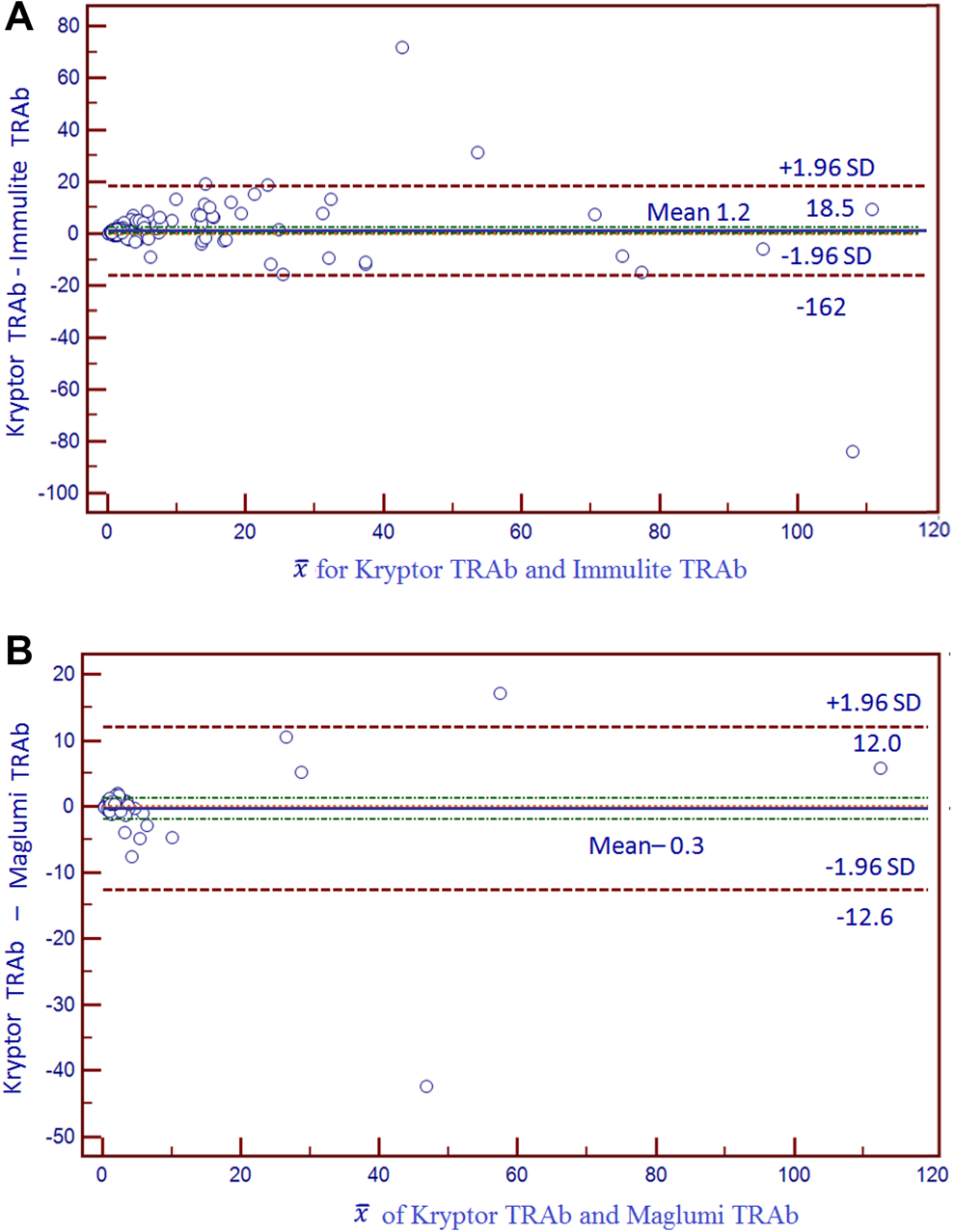
Bland-Altman plots. (A) Bland-Altman plot for Kryptor and Immulite. (B) Bland-Altman plot for Kryptor and Maglumi.

Table 2A shows the ICC obtained for each system, which all were >0.8. Table 2B displays the results obtained from comparison of means vs. the mean values obtained in each comparison. These results are shown in Figure 1A, B. Similar mean values were obtained on Kryptor and Maglumi, as compared to Kryptor vs. Immulite. Table 2C shows the level of agreement between the results obtained with the different systems. Quantitative results were converted into dichotomous qualitative variables (positive/negative). Kryptor and Immulite showed a slightly higher Kappa coefficient than Kryptor and Maglumi.

**Table 2:** Results of the analysis of concordance between the different methods for the totality of patients (n=194).

A	ICC	95%CI
Kryptor vs. Immulite	0.8618	0.8229 – 0.8927
Kryptor vs. Maglumi	0.9343	0.8933 – 0.9795

**B**	**Differences** **(** **x¯)**	**95%CI**
Kryptor vs. Immulite	1.2	−16 a +18
Kryptor vs. Maglumi	−0.3	−12 a + 12

**C**	**Kappa coefficient**	**95%CI**
Kryptor vs. Immulite	0.689	0.592 – 0.786
Kryptor vs. Maglumi	0.535	0.326 – 0.745

ICC, intra-class correlation coefficient.

In the comparative study of Kryptor vs. Immulite, the presence of constant and proportional bias was assessed on a Bland–Altman plot, which showed a difference between means (mD) of 1.2 as the absolute value.

Passing-Bablok regression analysis yielded the equation: *Y*=−0.7237 + 0.9152X, (X=Kryptor, Y=Immulite), with constant error (95%CI): −0.8349 to −0.5987 and a proportional error (95%CI): 0.7862 to 1.0387.

In the comparative study of Kryptor vs. Maglumi, Bland–Altman plot showed a difference between means (Dm) of −0.3 as absolute value.

Passing-Bablok regression yielded the equation: *Y*=−0.1600 + 1.0218X, (X=Kryptor, Y=Maglumi), with a constant error (95%CI): −0.7701 to 0.1621 and a proportional error (95%CI): 0.8571 to 1.3179.

The Pearson’s coefficients were r=0.78 for Kryptor vs. Immulite and r=0.76 K for Kryptor vs. Maglumi.

Table 3 displays the same calculations as in Table 2 applied to the subgroup of patients with GD. Passing-Bablok regression for this subgroup yielded the equation: *Y*=0.3557+1.368X, (X=Kryptor, Y=Immulite), with a constant error (95%CI) of 0.035–1.368 and a proportional error (95%CI) of 1.117–1.574. Passing-Bablok regression yielded the equation: Y=0.3913+0.9469X, (X=Kryptor, Y=Maglumi), with a constant error (95%CI) of −0.2311 to 1.0073 and a proportional error (95%CI) of 0.5797–1.1925.

**Table 3:** Analysis of concordance between the three methods for patients with Graves’ disease (n=113).

A	ICC	95%CI
Kryptor vs. Immulite	0.923	0.883 – 0.948
Kryptor vs. Maglumi	0.982	0.965 – 0.991

**B**	**Mean difference ** **(**x¯**)**	**95%CI**

Kryptor vs. Immulite	2.6	−14 a + 19
Kryptor vs. Maglumi	0.2	−5.6 a + 6.1

**C**	**Kappa** **coefficient**	**95%CI**

Kryptor vs. Immulite	0.586	0.44 – 0.732
Kryptor vs. Maglumi	0.15	0.11 – 0.40

ICC, intra-class correlation coefficient.

Indicators of diagnostic validity of the three systems for the detection of GD are shown in Table 4. Immulite showed the highest sensitivity and NPV, as compared to Kryptor and Maglumi, whereas Kryptor had the highest specificity. Similar PPV were obtained for Kryptor and Immulite, which were higher than in comparison with Maglumi.

**Table 4:** Diagnostic validity of the three methods.

	Kryptor	(95%CI)	Immulite	(95%CI	Maglumi	(95%CI
n	194		194		62	
Sensitivity, %	67	(58–74)	81	(74–88)	58	(42–73)
Specificity, %	80	(69–88)	73	(61–82)	53	(29–75)
PPV, %	85	(77–91)	84	(76–90)	73	(56–87)
NPV, %	57	(47–66)	69	(58–79)	36	(19–56)
LR+	3.42	(2.14–5.47)	2.99	(2.06–4.34)	1.23	(0.72–2.1)
LR−	0.41	(0.32–0.54)	0.25	(0.17–0.37)	0.8	(0.46–1.38)
AUC	0.81	(0.75–0.86)	0.86	(0.81–0.9)	0.54	(0.45–0.65)

95%CI, 95% confidence interval; PPV, positive predictive value; NPV, negative predictive value; LR+, positive likelihood ratio; LR−, negative likelihood ratio; AUC, area under the ROC curve.

The areas under the curve obtained for Kryptor and Immulite were similar and higher than for Maglumi, with the best cut-off points being 1.71 IU/L (*S*=80% and *Sp*=82%), 1.42 IU/L (*S*=73% and *Sp*=89%) and 1.48 IU/L (*S*=61 and *Sp*=55%), respectively.

## Discussion

The objective of this study was to determine whether the results of the three systems were superimposable, and explore indicators of diagnostic validity of the different TRAb assay methods both, for all the study population and for the subgroup of patients with GD.

Mean differences across the different TRAb assay methods studied were obtained. Passing-Bablok regression analysis was performed. Data was analyzed based on statistical criteria [20], [21], given the absence of data available in the literature about the biological variability of the parameter under study. According to the results obtained from the analysis of differences between both Kryptor vs. Immulite and between Kryptor vs. Maglumi, the 0 value was included in the confidence interval for mean differences; therefore, there were no statistically significant differences between the results obtained from the three analyzers (p≥0.05). This means that results would be interchangeable and the three analyzers can be considered a single virtual analyzer.

Hence, the results of Passing-Bablok regression analysis show that the 95%CI intercept in the origin does not contain 0, which indicates a constant difference; the 95%CI of the slope contains 1, which means that there were no proportional differences between the two analyzers. However, comparative study of Kryptor vs. Maglumi shows that the 95%CI intercept in the origin contains 0, which indicates the absence of constant differences. Conversely, the 95%CI of the slope does not contain 1, which means that there were not proportional differences between the two analyzers.

In the subgroup of patients with GD, comparison of Kryptor vs. Immulite shows a constant and a proportional error, whereas comparison of Kryptor vs. Maglumi does not show either a constant or proportional error. Of note, a lower number of samples were analyzed on Maglumi, as compared to the other methods, as it is detailed below in the limitations of the study. As a result, the confidence interval of the slope is very wide which indicates high imprecision. Finally, a very low Kappa coefficient was obtained for Kryptor and Maglumi.

We identified two types of results. On the one hand, the Bland–Altman plot shows that results of the three systems are superimposable. Good intra-class correlation coefficients were also obtained, exceeding 0.8. Nevertheless, visual inspection of paired data, plots, and results of Passing-Bablok regression analysis, and the level of agreement assessed by Kappa coefficient do not confirm that results are superimposable, and detect the presence of biases. In the light of such inconsistency, we cannot conclude that results are superimposable, and new reference values or cut-off points should be employed for other assay systems.

As to the results of regression analysis for Kryptor vs. Immulite, the intercept doubles in the total population, as compared to the GD subgroup, and the slope is closer to 1 in the total population than in the GD subgroup. Indeed, the slope is higher for this subgroup (1.368). This can be explained by the fact that the reagent used in Immulite only measures thyroid-stimulating antibodies [22]. Moreover, this would explain the higher difference observed at low values, and that the cut-off point suggested by Immunlite manufacturer is notably lower than the other cut-off points [14], [22].

As to the diagnostic validity, very similar results were obtained for Kryptor and Immulite, with a sensitivity and specificity of the same order, with Immulite having slightly better sensitivity and Kryptor having slightly better specificity. Similar results were also obtained from these analyzers concerning LH+ which was slightly higher for Kryptor, whereas the area under the ROC curves was slightly better in Immulite. In contrast, Maglumi yielded lower counts than the other analyzers in relation to TRAb. We are aware that the TRAb assay is not used for clinical diagnosis of GD, at least, in the strictest sense [17], [23], [24], [25], [26]. TRAb helps establish the etiological diagnosis of the disease. Apart from a cross-comparison of the three assay systems (primary objective of the study), we also assessed their diagnostic capacity.

In a study involving 124 patients with recently-diagnosed naïve hyperthyroidism, Scapatizo et al. [27] reported that Kryptor and Immulite had a good diagnostic performance. In another recent study [14] involving 383 patients (72 with GD, 55 with thyroiditis, 36 with multinodular goiter, 100 with extrathyroideal autoimmune diseases and 120 control subjects), Immulite showed a sensitivity of 100% and a specificity of 98%, with a cut-off value of 0.54. These results are not consistent with the ones obtained in our study (1.42), are far from those reported by Scapatizo et al. [27], and very close to those reported by the manufacturer. This method showed a good level of agreement with the Cobas/Elecsys (Roche) and the TRACK RIA system (Brahms, Thermo Fisher). The values obtained in that study exceed ours, probably as a result of the fact that our patients were treatment naïve.

The main limitation of this study is that a selection bias may result from the fact that we only included samples with a TRAb determination request. This way, only patients with a higher suspicion of GD were included in the study. Hence, higher validity values may be obtained for the parameters studied, as a consequence of the selection criteria employed.

Another potential limitation is that patients had different stages of GD and were at different points of treatment. At diagnosis, all patients had hyperthiroidism, whereas their functional status may change to hypothyroidism or normal function during the course of the disease. The variety of functional status must be understood in the context of a retrospective study.

Furthermore, the sample size is a strength for cross-comparison studies, as guidelines recommend that at least 40 patients be included. However, the number of samples compared with the Maglumi analyzer (n=62 for the total population and n=32 for the GD subgroup) was lower, as compared to comparison of the other two analyzers (n=194). Notably, a robust, widely-accepted gold-standard method was used for the analysis of diagnostic validity in the totality of the patients included in the study [28].

In conclusion, the three systems showed a good diagnostic performance for the identification of GD, with the Kryptor method having the highest specificity and PPV, and Immulite the one with the best sensitivity and NPV. The results of the three systems can be superimposed on Bland–Altman plot. However, Passing-Bablok regression analysis and agreement, as measured by Kappa coefficient, show that results are not interchangeable and demonstrates the presence of biases.
